# An open chat with… Sandro Sonnino

**DOI:** 10.1002/2211-5463.13689

**Published:** 2023-08-22

**Authors:** Ioannis Tsagakis, Sandro Sonnino

**Affiliations:** ^1^ FEBS Open Bio Editorial Office Cambridge UK; ^2^ Department of Medical Biotechnology and Translational Medicine University of Milano Italy

## Abstract

Sandro Sonnino is one of the founding members of the Editorial Board of *FEBS Open Bio*, having joined in 2011. He is also a member of the Editorial Board of *FEBS Letters* and is the Editor‐in‐Chief of *Glycoconjugate Journal*. He is full professor of biochemistry in the School of Medicine at the University of Milan, where he was also formerly coordinator of the Interdisciplinary Laboratory of Advanced Technology (LITA) and Director of the Department of Medical Chemistry, Biochemistry, and Biotechnology. His research is focused on the metabolism and biochemical properties of gangliosides and glycosphingolipids, and their role in cell signaling and the nervous system. He is a guest editor of this special “In the Limelight” issue on glycosphingolipids in disease, which features multiple Reviews and an original research article related to this field. In this interview, Sandro Sonnino discusses the ongoing importance of research on glycosphingolipids and his personal career journey.

## Graphical abstract

Sandro Sonnino, one of the founding members of the Editorial Board of *FEBS Open Bio*, is full professor of biochemistry in the School of Medicine at the University of Milan. He is a guest editor of this special “In the Limelight” issue on glycosphingolipids in disease; here, he discusses the ongoing importance of research on glycosphingolipids and his personal career journey.

## You are guest editor of this special “In the Limelight” issue on glycosphingolipids in disease. Why are glycosphingolipids so important?

For many years, glycosphingolipids were considered structural components of our membranes. But with progressive knowledge, they attracted many scientists who studied their implications in cellular processes. Glycosphingolipid abundance in the nervous system, particularly in the central nervous system, paved the way for neurochemical research and their involvement in important cellular processes became clear. Thus, the concept that glycosphingolipids, and particularly gangliosides, are necessary for the activity of membrane receptors and membrane enzymes was consolidated. Gangliosides work as a starting switch for the transduction of information through the plasma membrane or for the initiation of functional processes and are associated with several pathologies.

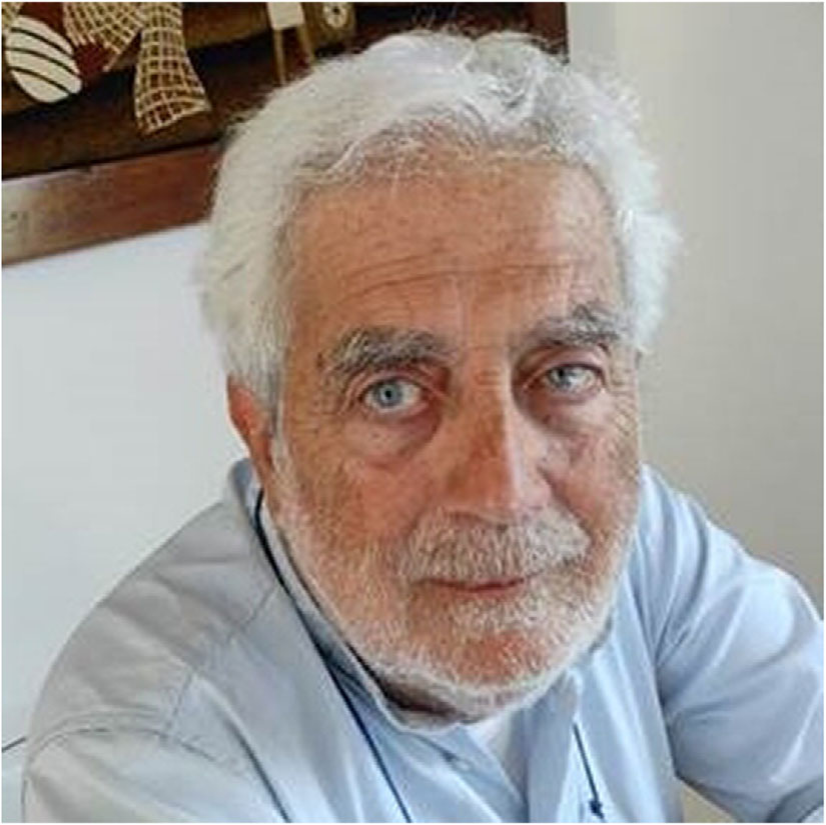

## What are the highlights of this issue?

In this special issue, we collected a few manuscripts from outstanding scientists, reporting on the specific role of the correct ganglioside quantities and their topography in relationship with the occurrence of diseases.

GM1 is one of the major gangliosides of the central nervous system but it is present in any tissue where its synthesis is regulated by specific glycosyltransferases. For unknown reasons, with aging, some of these enzymes are less expressed, which progressively reduces the GM1 ganglioside content in turn affecting the following processes necessary for cell signaling and cell physiology. The result is the occurrence of clinical symptoms that in the majority of cases are associated with the normal age‐related decline of body and mind. But in some cases when the acceptable ganglioside content is below a certain threshold, cells, and neurons in particular, progressively degenerate and inflammatory processes occur.

Hence, ganglioside topography is associated with ganglioside quantity and with ganglioside segregation in lipid membrane domains, the lipid rafts, and a small area inside the domain itself. Membrane organization deriving from the ganglioside content seems to be very important in enabling or preventing the interaction with membrane receptors and membrane enzymes. This is also very important for hormonal release and availability. For example, hormones can in some cases be synthesized but not correctly released, which could translate into a breakdown between ligand–receptor interactions.

This could be a direct consequence of a membrane that is not organized correctly, or it could also be due to new membrane–membrane interactions deriving from altered ganglioside organization. Also, it is worth bearing in mind the carbohydrate head‐to‐head interactions that have a direct implication on subcellular fusions and can impede maintenance of separation of subcellular fractions.

The first Review article in this issue, authored by myself, describes the role of GM1 in neurons and how its depletion may contribute to the onset of Parkinson's disease [1]. This leads into the second Review article, authored by Fokion Spanos and Michela Deleidi, which discusses how changes in glycolipid metabolism can cause inflammatory reactions and contribute to Parkinson's disease and other neurodegenerative diseases [2]. Tatsuro Mutoh and colleagues then describe how disruptions in glycolipid and sphingolipid metabolism are observed in cases of encephalomyeloradiculoneuropathy (EMRN), which affects the brain, spinal cord, and peripheral nerves, and the involvement of such molecules in the immune system [3]. Concluding the section on neurodegenerative diseases, Alessandra d'Azzo and colleagues describe how ectopic accumulation of aberrantly processed/degraded glycosphingolipids can affect the topology of membrane contact sites, resulting in neurodegeneration [4]. Glycosphingolipids also play roles in other diseases, including cystic fibrosis and potentially some cases of male infertility; Massimo Aureli *et al*. consider the role of sphingolipids in the homeostasis of cystic fibrosis transmembrane conductance regulator (CFTR) and the potential of manipulating sphingolipid metabolism to ameliorate cystic fibrosis [5]. Koichi Furukawa *et al*. [6] describe their unexpected discovery that GM2/GD2 synthase knockout mice exhibit aspermatogenesis, and the implications for male infertility in humans.

The aforementioned Review articles all address the role of host sphingolipids in disease, but in the case of parasite diseases, the sphingolipids of the infectious agent also need to be considered; in this issue, Richard D. Cummings provides a detailed overview of the glycosphingolipids in various parasites of humans, as potential targets for new drugs and diagnostics [7]. In the final Review article in this collection, Jacques Fantini examines the full breadth of human diseases that involve gangliosides, before proposing a new classification system for ganglioside‐binding domains of proteins to aid rational design of new drugs [8]. In addition to these excellent Review articles, I am delighted to be able to include in this issue an original research article by Robert Ledeen and colleagues, which reports that application of GM1 can ameliorate memory and movement disorders in mice lacking both copies of the GM3 synthase gene, suggesting the therapeutic potential of synthetic GM1 [9].

## How did you become interested in studying lipids?

I started studying glycosphingolipids in 1972, when I was accepted to carry out my thesis on gas chromatographic analyses of gangliosides by the Institute of Biochemistry in the School of Medicine, University of Milan. My mentor, Professor Guido Tettamanti, and all the laboratory staff were studying gangliosides and the enzyme sialidase. A new procedure for laboratory preparation of gangliosides was being developed by Prof. Tettamanti and colleagues. A lot of known and unknown gangliosides were available. I obtained my MSc in chemistry and Professor Tettamanti asked me to remain in the laboratory as a young scientist to continue research in the field of ganglioside chemistry. I accepted and started my studies on gangliosides.

## What are some recent key findings in the area of glycosphingolipids, and what are their implications for the field?

Gangliosides are glycosphingolipids containing one or more residues of a negatively charged sialic acid. The molecule is amphiphilic, being formed of a lipid moiety called ceramide and a hydrophilic chain. The main finding related to gangliosides is the possibility to use their free oligosaccharides as drugs for treatment of neurodegenerative diseases. The free oligosaccharide is the sole carbohydrate portion of a ganglioside, and it mimics the properties of their corresponding gangliosides, but contrary to these, they can cross the blood brain barrier [1].

## Why is studying membrane lipids important? How has the field of lipid biology helped other fields?

In cell biology, it is important to understand the role played by membrane organization and composition. Gangliosides have been linked to aberrant glycosylation and cancer [10]. The membrane ganglioside content is determined by complex genetic processes and must remain constant to maintain the necessary membrane organization. This is necessary to allow the correct ganglioside–protein and ganglioside–ganglioside interactions considered as the switch for the signaling that governs cellular functions. Cancer proliferation in many cases is due to abnormal glycosylation processes that prevent the cell–cell recognition necessary for signaling to the nucleus.

Other studies connected an incorrect neuronal content of gangliosides to neurodegeneration [11]. Neuronal degeneration in many cases is due to genetic repression of the glycosyltransferases necessary for the synthesis of gangliosides and their correct distribution in neuronal membranes. This could prevent many signaling pathways related to neurotrophine receptors, thus highlighting how lipid biology influences key biological processes.

## How do you see the field of lipid biology or lipid rafts developing in the future?

Glycobiology is a complex discipline, not well‐understood when not related to starch, glucose, and diabetes. Important results require long studies and many steps before acceptance. As a result, the field of lipid biology is not developing very well, due to progressive reduction of group studies in a field that is not well‐funded.

## Could you give us an example of how discoveries in lipid biology have been translated for use in the clinic?

I recall that gangliosides were used for therapy of peripheral neuropathies for 20 years and injected in several millions of peoples all over the world. They were then removed due to a possible association with Guillen–Barrè syndrome. Guillen–Barrè syndrome is the production of autoantibodies that recognize several glycans linked to membrane lipids and proteins belonging to the peripheral nervous system. These autoantibodies are released after infection of bacteria or virus having a similar cell surface chain to those present on the human membrane. While a link to Guillen–Barrè syndrome was later disproven, the field has since been marked and social stigma remains. The fact that gangliosides were prepared by extraction from calf brains with the necessity to use highly controlled animals, and the inability to produce them by chemical synthesis contributed to their removal as a treatment. The use of ganglioside oligosaccharides, which could be prepared by specific bacterial engineering could reignite industrial interest. Gangliosides, as amphiphilic compounds, are soluble in water only as micelles over nanomolarity, and they are not useful if injected when it is required that they reach the central nervous system. The oligosaccharide chain of gangliosides maintains the same interaction properties expressed by gangliosides with proteins, enzymes, and receptors, but are able to cross the blood brain barrier and reach central neurons.

## From a personal standpoint, which award in recognition of your work are you most proud of?

Actually I am president of the International Glycoconjugate Organization, Editor‐in‐Chief of *Glycoconjugate Journal* and reviewer for ganglioside papers submitted to the most important scientific journals.

## Have you acted as a mentor to early career researchers? What qualities/traits are essential for being a successful mentor?

I was mentor of several scientists, now full or associate professors of biochemistry. It is necessary to know the field well, to be a good teacher and to be frank and loyal.

## What is your proudest achievement outside science?

I have been married for 50 years, I have a daughter and a grandson. I have a good family.
